# MicroRNA-221-3p Suppresses the Microglia Activation and Seizures by Inhibiting of HIF-1α in Valproic Acid-Resistant Epilepsy

**DOI:** 10.3389/fphar.2021.714556

**Published:** 2021-08-23

**Authors:** Meng Fu, Yiqing Zhu, Junqi Zhang, Wei Wu, Yunxia Sun, Xuemei Zhang, Jie Tao, Zhiping Li

**Affiliations:** ^1^ Department of Clinical Pharmacy, Children’s Hospital of Fudan University, National Children’s Medical Center, Shanghai, China; ^2^ Academy of Chinese Medical Sciences, Zhejiang Chinese Medical University, Hangzhou, China; ^3^ Department of Pharmacology, School of Pharmacy, Fudan University, Shanghai, China; ^4^ Central Laboratory, Putuo Hospital, Shanghai University of Traditional Chinese Medicine, Shanghai, China

**Keywords:** valproic acid-resistant epilepsy, microRNA-221-3p, hypoxia-inducible factor-1α, microglia, neuroinflammation

## Abstract

One-third of patients with epilepsy suffer from drug-resistant epilepsy (DRE). Valproic acid (VPA) is a classic anticonvulsant drug, and its resistance is a crucial predictor of DRE, but the pathogenesis remain unknown. Most patients with VPA-resistant epilepsy appear distinct inflammatory response and local hypoxia. Hypoxia-inducible factor (HIF)-1α is an essential effector molecule of hypoxia and inflammation, and may exert therefore a significant effect on the development of VPA-resistant epilepsy. We systematically assess the significance of HIF-1α on children and mice with VPA-resistant epilepsy, and investigated the micro (mi) RNAs that regulate HIF-1α expression. We established models of VPA-sensitive epilepsy and VPA-resistant epilepsy in mice, and confirmed that they had significant differences in epileptic behavior and electroencephalography data. Through proteomics analysis, we identified that HIF-1α was overexpressed in mice with VPA-resistant epilepsy, and regulated the expression of interleukin-1β and tumor necrosis factor-α. Increased expression of HIF-1α led to the increase of microglia and induced their polarization from the M2 phenotype to M1 phenotype, which triggered the release of proinflammatory mediators. Bioinformatics analysis of public databases demonstrated that miR-221-3p was reduced in VPA-resistant epilepsy, and negatively regulated HIF-1α expression. Intervention using miR-221-3p mimics reduced HIF-1α expression markedly and suppressed the activation of microglia and the release of inflammatory mediators, which relieved epileptic seizures of VPA-resistant epilepsy. These observations reveal miR-221-3p/HIF-1α as essential component in pathogenesis of VPA-resistant epilepsy which represent therapeutic antiseizure targets.

## Introduction

Epilepsy is a chronic brain condition caused by sudden abnormal discharge from brain neurons. Epilepsy affects >70 million people worldwide (Thijs et al., 2019). Treatments of epilepsy include medication, surgery and ketogenic diet, among which medication is the main approach (Kanner et al., 2018). Anti-epileptic drugs (AEDs) could inhibit 60–70% seizures. However, 30–40% of patients with drug-resistant epilepsy (DRE) cannot control seizures effectively through AEDs (Kalilani et al., 2018). Valproic acid (VPA) is a classic AEDs (Chen et al., 2018). Clinical evidence suggests that VPA resistance is a vital predictor of DRE (Gesche et al., 2017), but the mechanism of VPA-resistant epilepsy remained largely elusive. Patients fail to attain a seizure-free lifestyle, which increases the risk of psychosocial dysfunction, injuries, and premature death (Löscher et al., 2020).

Numerous studies have strongly supported the role of inflammation in DRE pathophysiology (Weidner et al., 2018; Fu et al., 2020; Ouédraogo et al., 2021). Multifarious inflammatory molecules and signaling pathways have been shown to influence the outcome of experimental models and patients with DRE (Weidner et al., 2018).

Microglia are the resident immune cells in the brain. Microglia are universally involved in the response to diverse forms of insults and diseases in the central nervous system (CNS) (Patel et al., 2019). Studies have shown that microglia are activated in the brain of patients with DRE (Devinsky et al., 2013). Pioneering studies have indicated that proinflammatory factors such as interleukin (IL)-1β, tumor necrosis factor (TNF)-α, High mobility group box (HMGB)1, cyclooxygenase (COX)-2, and prostaglandin (PG)E2 have increased significantly in the epileptic foci and blood of patients with DRE (Rana and Musto, 2018). Simultaneously, expression of interleukin-1 receptor (ILR)1, and toll-like receptors (TLRs) increase correspondingly, resulting in activation of inflammation-related signaling pathways such as IL-1β/ILR1, HMGB1/TLR4 (Strauss and Elisevich, 2016). Previously, we found that expression of IL-1β, chemokine (C-C motif) ligand (CCL)3, TNF-α, HMGB1 and other inflammatory mediators was increased greatly in the blood samples of children with VPA-resistant epilepsy (Wang and Li, 2019; Fu et al., 2020). These data suggested that neuroinflammation may play a crucial part in the pathologic mechanism of VPA-resistant epilepsy.

Moreover, DRE patients are characterized by local hypoxia in the brain, which is caused by rapid consumption of oxygen and vasoconstriction due to repeated seizures (Bateman et al., 2008). Hypoxia-inducible factor (HIF)-1 is an endogenous transcription factor that contributes to the cellular response to hypoxia. HIF-1 is a heterodimer that consists of constitutively expressed HIF-1β and HIF-1α subunits (Balamurugan, 2016). If oxygen is available, HIF-1α is degraded by oxygen-dependent prolyl hydroxylation. If the oxygen concentration drops, a stable HIF-1 complex is formed, which activates the transcription of several genes encoding proteins involved in angiogenesis, glucose metabolism, and cell proliferation/survival (Semenza, 2003; 2014). Notably, HIF-1α induces the transcription of inflammation-related proteins such as T-cell immunoglobulin and mucin-domain containing-3 and vascular endothelial growth factor in various diseases. Studies have shown that HIF-1α is involved in microglia activation in patients with Alzheimer’s disease (Baik et al., 2019). However, the contribution of HIF-1α in VPA-resistant epilepsy remain unknown.

MicroRNAs (miRNAs) are a class of endogenous noncoding single-stranded RNA molecules of size 22–25 nt (Lu and Rothenberg, 2018). MiRNAs are major players in posttranscriptional gene regulation in diverse biological processes (Fabian and Sonenberg, 2012). Multifarious specific miRNAs exist in different brain regions and different types of brain cells (O'Carroll and Schaefer, 2013). Various reports have suggested miRNAs to be functional regulators of epileptogenesis (Henshall et al., 2016). Different miRNAs, including miR-132 (Korotkov et al., 2020), miR-34a (Organista-Juárez et al., 2019), and miR-146a (Aronica et al., 2010) appear to be actively involved in DRE pathogenesis. As noted above, miRNAs may play crucial roles in the regulation of inflammation in patients with VPA-resistant epilepsy.

In this study, we assess the significance of HIF-1α in VPA-resistant epilepsy. We sought to identify the miRNA that regulates HIF-1α expression in a mouse model and in patients with VPA-resistant epilepsy.

## Methods

### Patients Samples

The present study recruited 24 children suffering from epilepsy (0–18 years) from the Children’s Hospital of Fudan University. Sixteen cases were resistant to antiepileptic drugs, including VPA (Supplementary Table S1), and eight cases were sensitive to VPA (Supplementary Table S2). The diagnosis of DRE was based on criteria published by the International League Against Epilepsy (ILAE) criteria (Kwan et al., 2010). Briefly, children with DRE failed to be free of seizures after ≥2 tolerated regimens of antiepileptic drugs, including VPA. For children with drug-sensitive epilepsy, seizures disappeared after using VPA for ≥6 months, and there are no epileptiform discharges in the electroencephalogram (EEG) after treatments. The exclusion criteria were patients with: 1) severe adverse reactions to drugs; 2) poor compliance with use of antiepileptic drugs; 3) unreliable record of seizure frequency; 4) history of pseudo-seizures; 5) drug abuse; 6) malignant diseases (e.g., brain tumors, metastasis); 7) hepatic or renal failure. The study protocol was approved by the Ethics Committee of the Children’s Hospital of Fudan University (Shanghai, China). Written informed consent was obtained from all patients and healthy controls included in our study.

Approximately 2 ml of peripheral blood was collected from each enrolled individual. Then, the plasma samples were obtained by centrifugation and stored at −80°C. Hemolytic plasma samples were excluded.

### Animals

C57BL/6 male mice were used for the study (*n* = 98). All mice were social housed under standardized conditions of light, temperature and humidity, environmental enrichment, and had access to food and water ad libitum. All possible efforts were made to minimize animal suffering and the number of animals used. This study was carried out in accordance with the principles of the Basel Declaration. The protocol was approved by the Institutional Ethics Committee of Children’s Hospital of Fudan University.

### Creation of a Kainic Acid-Induced Model of Chronic Epilepsy

A KA-induced temporal lobe epilepsy (TLE) model was created in mice, as described previously. Briefly, all mice were fed for 1 week to adapt to the environment, and then were assigned randomly into an epilepsy group or control group. Seizures were induced by administration of KA (3 mg/ml, 0.2 ml/mouse, i.p.) dissolved in physiologic (0.9%) saline. Mice in the control group were given an equal amount of 0.9% saline. All mice were monitored for 1 h after KA injection to evaluate seizures using the Racine Scale (Racine, 1972). Status epilepticus (SE) was defined as an epileptic seizure of grade three or greater lasting longer than 30 min. Seizures were terminated at 1 h after onset with the use of sodium pentobarbital, if necessary.

### Pharmacological Assessment

Four weeks after KA treatment, the epileptic behavior of mice was scored. Twelve epileptic mice were selected as the model group, and the number of seizures was not significantly different from that of other mice. The remaining mice were given VPA (150 mg/kg, p.o.; Sanofi, Paris, France) twice-daily for 4 weeks. Studies have shown that this dose is an efficacious therapeutic dose of VPA for epileptic mice. Mice in the control group and model group was given solvent of VPA. After the final administration, mice were housed individually and their epileptic behavior and EEG data recorded. The latter was carried out according to the stereoelectroencephalography approach in which intracerebral multiple contact electrodes (AP: −2.2 ± 0.1 mm, ML: +0.8 ± 0.1 mm, DV: −1.4 ± 0.1 mm) were employed (Bartolomei et al., 2012). Seizures were characterized by synchronized high-voltage amplitude oscillations. EEG data and behavior data were reviewed by two trained experimenters blinded to the experimental conditions to identify motor seizures. Finally, of the mice given VPA, 12 mice with the best treatment effect were placed in VPA-sensitive group and 12 mice with the worst treatment effect were placed in the VPA-resistant group.

### Tissue Preparation

Mice were decapitated with isoflurane for proteomics analysis, western blotting, and real-time reverse transcription-quantitative polymerase chain reaction (RT-qPCR). Brain tissues were removed rapidly and placed in ice-cold phosphate-buffered saline (PBS). The dissected hippocampus was snap-frozen in liquid nitrogen and stored at −80°C. For histology, mice were anesthetized and underwent perfusion with 50 ml of saline, followed by 80 ml of 4% paraformaldehyde. Brains were removed and fixed in 4% paraformaldehyde overnight at 4°C. After sequential dehydration with 20 and 30% sucrose for 48 h, coronal sections of thickness 10 μm were cut using a freezing microtome and then attached to slides. These hippocampal sections were stored at 4°C for Nissl staining and immunofluorescence staining.

### Proteomics Analysis

Total protein was extracted from the hippocampus of mice in the VPA-resistant epilepsy group and VPA-sensitive epilepsy group using SDT lysis buffers. The protein concentration was measured by a bicinchoninic acid (BCA) kit (Beyotime Institute of Biotechnology, Shanghai, China). Then, supernatant proteins were digested in trypsin, as described previously. The filter-aided sample preparation method was used for sample purification, chemical derivatization, and enzymatic digestion. After protein digestion, peptides were labeled according to the operation instructions of the Tandem Mass Tag kit (Thermo Fisher Scientific, Waltham, MA, United States ). Labeled peptides from each group were mixed equally and then fractionated using the High pH Reversed-Phase Peptide Fractionation kit (Thermo Fisher Scientific). Each sample was desalted and passed into a trap column for gradient elution. Each eluted peptide sample was dried by vacuum centrifugation for high-performance liquid chromatography (HPLC). Each fractionation sample was separated by the Easy nLC 1,200 system (Thermo Fisher Scientific) at a nanoliter flow rate. Eluent A contained 0.1% (v/v) formic acid in water. Eluent B contained 84% acetonitrile and 0.1% formic acid in water. Mass spectrometry data were acquired with a Q-Exactive mass spectrometer (Thermo Fisher Scientific). With respect to parameters: the scanning range of the precursor ion was mass/charge (m/z) 300–1800; full scans were acquired at a resolution of 70,000 at m/z 200; dynamic exclusion was set at 60.0 s; the target for automatic gain control was 1e6; the maximum IT was 50 ms. Twenty fragment maps were collected after each full scan to obtain the m/z of peptides and peptide fragments; the type of MS2 activation was HCD; the isolation window was m/z 2; tandem mass spectrometry scans were acquired at a resolution of 35,000 at m/z 200; normalized collision energy was 30 eV; underfill was 0.1%.

### Gene Ontology and Kyoto Encyclopedia of Genes and Genomes

Annotation of the proteome was undertaken based on the Gene Ontology (GO) database (http://geneontology.org/) using Blast2GO, which comprises four steps: blast, mapping, annotation, and annotation augmentation. Proteins were classified by GO annotation based on three categories: biological process (BP), cellular component (CC), and molecular function (MF). Enrichment of signaling pathways was identified based on the Kyoto Encyclopedia of Genes and Genomes (KEGG) database (www.genome.jp/kegg/pathway.html/) using the KEGG Automatic Annotation.

### Construction and Analysis Protein-Protein Interaction Network

The Search Tool for the Retrieval of Interacting Genes/Proteins (STRING; http://string-db.org/) database was used to analyze the interaction of hub genes and to construct a PPI network (Szklarczyk et al., 2015). The latter was visualized by Cytoscape (http://cytoscape.org/) (Su et al., 2014) and the MCODE plugin was used to select the hub clustered subnetworks of highly intraconnected nodes with the default parameters (degree cut-off ≥ 2, node score cut-off ≥ 0.2, K-core ≥ 2, and maximum depth = 100).

### Western Blotting

Western blotting was employed to verify the results of proteomics analysis. Samples of hippocampal tissues were homogenized and centrifuged. The protein concentration was determined by the BCA protein assay kit (Beyotime Institute of Biotechnology) and adjusted to 2 μg/μl. Proteins (8 μl) were separated by sodium dodecyl sulfate–polyacrylamide gel electrophoresis using 10% gels, and then transferred to polyvinylidene difluoride (PVDF) membranes. The latter were blocked with 5% nonfat milk in PBS for 1 h. Next, the blocked PVDF membranes were incubated with antibodies (all purchased from Abcam, Cambridge, United Kingdom) against HIF-1α (1:200 dilution), anti-IL-1β (1:1,000), anti-TNF-α (1:1,000), anti-cluster of differentiation (CD)86 (1:1,000), and CD206 (1:1,000) overnight at 4 °C. After washing with Tween-20 and Tris-buffered saline (TBST), PVDF membranes were incubated with a horseradish peroxidase-conjugated secondary antibody (1:1,000; Abcam). β-actin (1:1,000; Abcam) was used as an internal control. Gray values were measured using ImageJ (National Institutes of Health, Bethesda, MD, United States ). The relative expression of samples in different duplications were standardized by a same sample of control group.

### Nissl Staining

For observation of hippocampal neurons, Nissl staining was undertaken according to a method described previously. Briefly, the brain sections were washed with deionized water for 2 min, and then immersed in 1% toluidine blue (Yeasen, Beijing, China) at 37°C for 10 min. After rinsed in deionized water, slices were dehydrated in graded ethanol solutions. Slices were cover-slipped with neutral balsam.

### Immunofluorescence Staining

We wished to detect the number of neurons (using NeuN) and microglia (using ionized calcium-binding adaptor molecule (Iba)-1) and whether there was co-labeling of microglia and HIF-1α in the hippocampal tissue of mice with VPA-resistant epilepsy. Hence, immunofluorescence was undertaken using previously described methods with slight adjustment (Han et al., 2018). Briefly, frozen sections were permeabilized with 1% Triton X-100 and blocked with PBS containing 5% bovine serum albumin. Without washing, sections were incubated overnight with primary antibodies against NeuN, Iba-1, and HIF-1α. Following further washing, sections were incubated with goat anti-rabbit antibody conjugated with Alexa Fluor 594 (for NeuN or Iba-1) and goat anti-mouse antibody conjugated with Alexa Fluor 488 (for HIF-1α) for 1 h at 37 °C. Then, sections were rinsed in deionized water, and mounted with Vectashield™ with 4’,6-diamidino-2-phenylindole. Fluorescence images were captured using a Virtual/Digital Slice Microscope (Olympus, Tokyo, Japan). Cells with a distinct nucleus were counted by a pathologist who was blinded to the grouping of our study.

### Enzyme-Linked Immunosorbent Assay

Expression of IL-1β and TNF-α in plasma from children with VPA-resistant epilepsy or VPA-sensitive epilepsy was quantified by ELISA kits (Shanghai Enzyme-linked Biotechnology, Shanghai, China) according to manufacturer instructions.

### Bioinformatics Analysis

Profiling of hippocampal miRNA of two independent datasets was re-analyzed. Profile 1 was the GSE99455 dataset from Gene Expression Omnibus (www.ncbi.nlm.nih.gov/geo/query/acc.cgi?acc=GSE99455). Profile 2 was from the Supplementary Material of as study published by Kan and colleagues (https://link.springer.com/article/10.1007/s00018-012-0992-7) (Kan et al., 2012). Profile 1 included 16 patients with DRE and eight post mortem controls. Profile 2 included 20 DRE patients and 10 post mortem controls. Analyses of differential expression were undertaken using “DESeq2” in R (R Institute of Statistical Computing, Vienna, Austria) and adjusted *p* < 0.05 and |log2-fold change (FC)| >1.5 were the cutoff thresholds. The overlapping miRNA dataset was obtained by taking the intersection of the differentially expressed miRNA sets of Profile 1 and Profile 2. Subsequently, the miRNAs that regulate HIF-1α expression were searched for through the online database starbase (http://starbase.sysu.edu.cn/), which were verified in samples from children with VPA-resistant epilepsy or VPA-sensitive epilepsy. Simultaneously, the binding sites of miRNA and HIF-1α were detected using a luciferase reporter assay.

### Real-Time PCR Analysis

Expression of miRNAs, inducible nitric oxide synthase (iNOS), CD86, arginine (Arg)1 and CD206 in the hippocampus was detected using RT-qPCR. Total RNA extracted from the hippocampus was reverse-transcribed into complimentary-DNA using a FastQuant RT kit (Tiangen, Beijing, China). Primers were synthesized by Sangon Biotechnology (Shanghai, China). RT-qPCR was done in triplicate in a minimum of three independent experiments according to the instructions of the PCR kit (Tiangen). Relative gene expression was calculated by the 2−ΔΔCT method.

### Luciferase Reporter Assay

The predicted binding site of the miRNA in the target gene was verified using a luciferase reporter gene assay carried out in human embryonic kidney (HEK)-293T cells. Cells were placed in 24-well plates. The confluence reached 60–70% after 24 h of incubation. HIF-1A-3′-untranslated region (UTR) wild type (wt) and HIF-1A-3′-UTR mutant reporter plasmids were constructed in advance. Then, miRNA mimics and a mimic negative control (NC) were co-transfected with the luciferase reporter vector into HEK-293 T cells. The luciferase activity was analysed by dual luciferase assay system (Promega, Fitchburg, WI, United States ).

### Statistical Analyses

Statistical analyses were carried out using SPSS 20.0 (IBM, Armonk, NY, United States ). Data are the mean ± standard deviation (SD). The differences between two or among more groups were assessed via two-sided unpaired Student’s t-test (two groups) or one-way ANOVA followed by Fisher’s least significant difference post hoc test (multiple groups). *p* < 0.05 was considered significant. Analyses were undertaken in a blinded manner.

## Results

### Behavior and Electroencephalogram in Mice With VPA-Resistant Epilepsy

**FIGURE 1:**
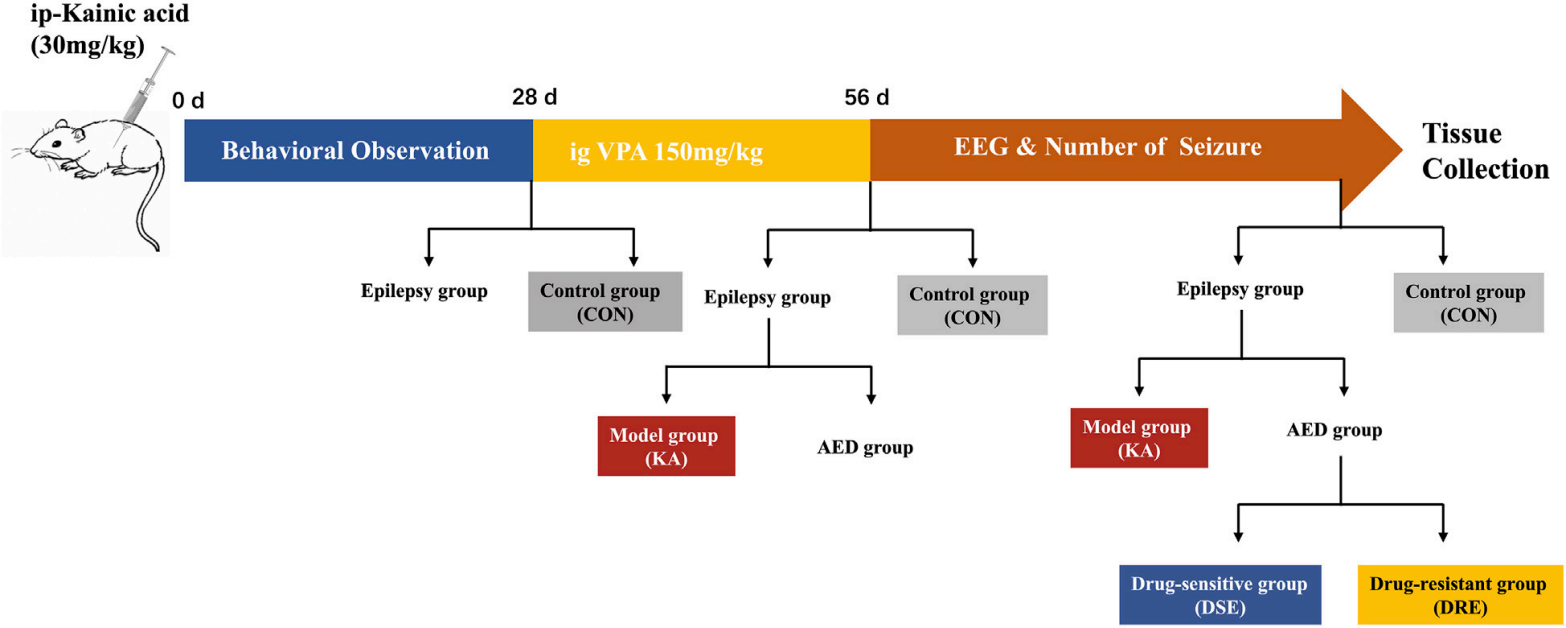
Schematic showing the creation of a VPA-resistant epilepsy model in mice. Step 1: Sixty mice with temporal lobe epilepsy were established by intraperitoneal administration of KA. Step 2: twenty epilepsy mice were assigned into epilepsy model, and 48 epilepsy mice were treated with VPA for 4 weeks. Step 3: After administration, the mice were divided into VPA-sensitive group and VPA-resistant group according to their epileptic behavior and EEG data.

Ninety-eight mice were used in this study (Figure 1): 12 mice were in the control group (CON), and 86 mice were given KA to induce TLE. Twelve of 86 mice died after SE; 14 of 86 were excluded because SE did not appear 2 h after KA injection. Thus, we obtained 60 epilepsy mice, of which 12 mice were assigned to the KA-induced epilepsy model (KA) and 48 mice were given VPA. After VPA administration, 12/48 mice with the best treatment effect were assigned to the VPA-sensitive group (DSE), and 12/48 mice with the worst treatment effect were assigned to the VPA-resistant group (DRE).

**FIGURE 2:**
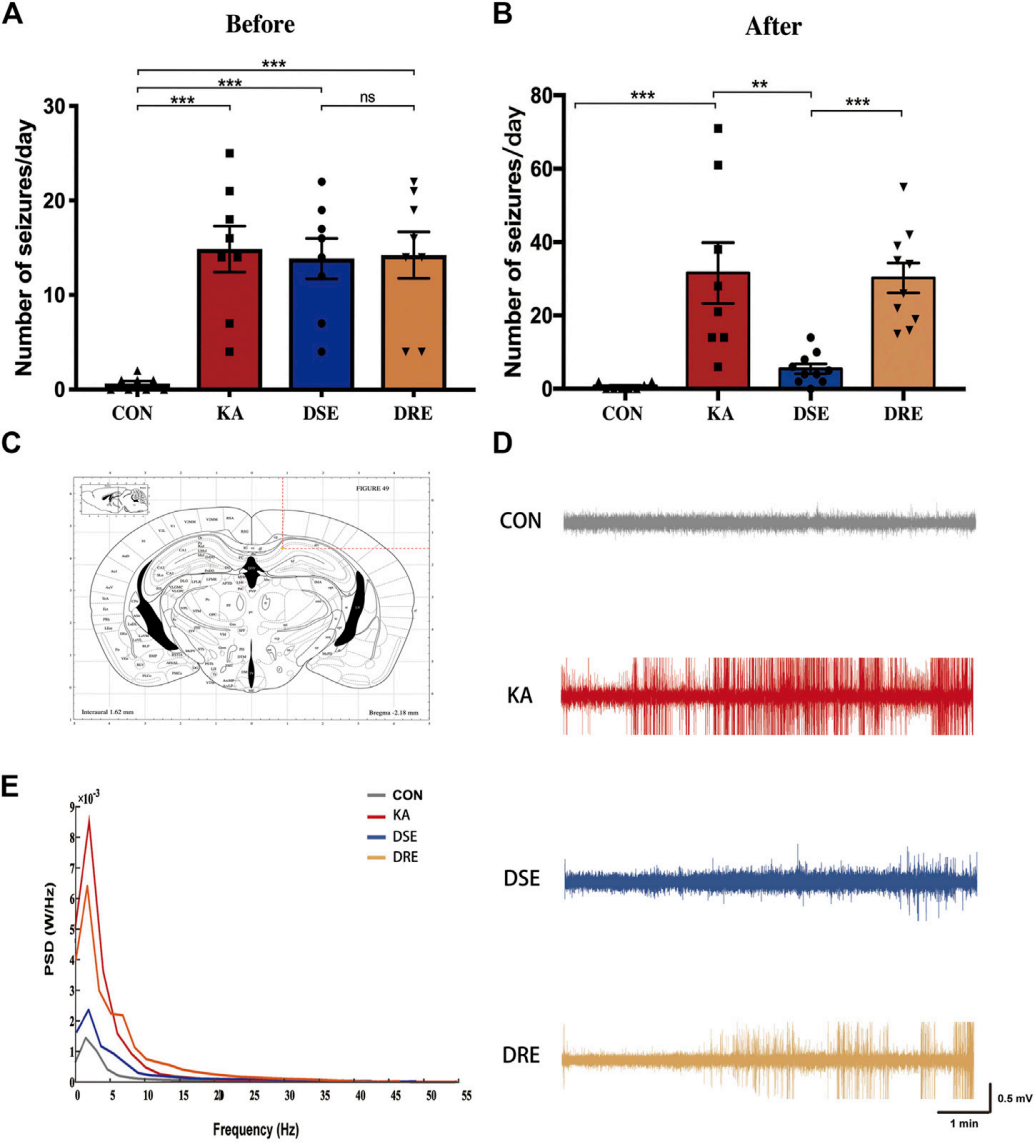
Data of EEGs and behavior in mice with KA-induced epilepsy (*n* = 12). **(A)** There is no obvious difference in number of seizures in mice with VPA-sensitive epilepsy and VPA-resistant epilepsy group before VPA administration. **(B)** After VPA administration, mice with VPA-resistant displayed increased seizure severity when compared to mice with VPA-sensitive epilepsy. **(C)** Location of EEG electrodes in mouse hippocampus: AP: −2.2 ± 0.1 mm, ML: +0.8 ± 0.1 mm, DV: −1.4 ± 0.1 mm. **(D)** The field potential signal from mice in control, epilepsy model, VPA-sensitive epilepsy, and VPA-resistant epilepsy groups. **(E)** Representative EEGs showing typical epileptiform discharges in mice with VPA-resistant epilepsy. **(F)** The power spectral density of delta band of the VPA-resistant group was higher than that of the VPA-sensitive group. Values are the mean ± S.D. Unpaired *t*-test. *p < 0.05, **p < 0.01, and ***p < 0.001. ns, not significant. CON: control, KA: KA-induced epilepsy, DSE: VPA-sensitive epilepsy, and DRE: VPA-resistant epilepsy.

The number of seizures was counted according to the Racine Scale. After SE and before VPA administration, there is no obvious difference in the number of seizures among the epilepsy model, VPA-sensitive epilepsy, and VPA-resistant epilepsy groups (Figure 2A). 4 weeks after VPA administration, the seizure frequency of mice with VPA-sensitive epilepsy was dramatically lower than that in the VPA-resistant epilepsy group (Figure 2B).

EEG data confirm that there was a difference in the seizure activity between VPA-resistant epilepsy and VPA-sensitive epilepsy (Figure 2C): EEG data were line with the epileptic behavior. Numerous epileptiform discharges with high-amplitude spikes and sharp waves were present in mice of epilepsy model group, which were ameliorated significantly in the VPA-sensitive epilepsy group. Notably, there were many high-amplitude rhythmic discharges in the VPA-resistant epilepsy group (Figure 2D).

In parallel, the power spectral density was computed for each EEG frequency band in accordance with the Welch method: delta (0–4 Hz), theta (5–8 Hz), alpha (9–12 Hz), beta (13–30 Hz) and gamma (31–100 Hz). The delta band of the model group was higher than that of the control group (Figure 2E). This phenomenon was reduced in the VPA-sensitive epilepsy group but not in the VPA-resistant epilepsy group. Taken together, these data demonstrated difference in seizures severity between the VPA-resistant group and VPA-sensitive group after VPA treatment.

### Proteomics Analysis

**FIGURE 3:**
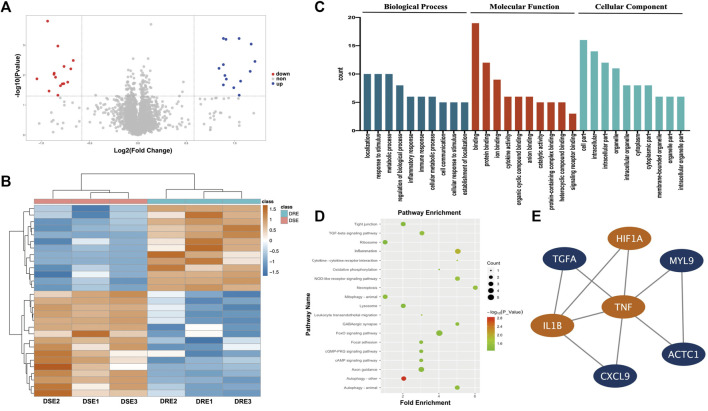
Quantitative proteomics analysis of the hippocampus in mice. **(A)** Volcano plot of differentially expressed protein in hippocampus between VPA-resistant epilepsy and VPA-sensitive epilepsy. Red dots are down-regulated proteins. Blue dots are up-regulated proteins. **(B)** Heatmap of 29 differentially expressed protein in hippocampus between VPA-resistant epilepsy and VPA-sensitive epilepsy. **(C)** The biological process, molecular function and functional enrichment analysis of the differentially expressed proteins between the VPA-sensitive group and the VPA-resistant group through the GO database. **(D)** Differentially expressed proteins are enriched in multiple inflammation-related signaling pathways using the KEGG database. **(E)** The top 1 connectivity module contained HIF-1α, IL-1β, and TNF-α in the PPI network of differentially expressed proteins. DSE: VPA-sensitive epilepsy, DRE: VPA-resistant epilepsy.

The abundance of hippocampal proteins was compared between the VPA-resistant epilepsy group and VPA-sensitive epilepsy group using liquid chromatography-tandem mass spectrometry. A total of 30,738 unique peptides were identified, which corresponded to 4,924 unique proteins after searching SwissProt-Rodentia protein database and annotated at a false discovery rate of 1% for peptides. The cutoff for differential abundance of proteins was FC > 1.5 (upregulated) or < 0.67 (downregulated) and *p* < 0.05. According to these screening criteria, 29 proteins exhibited significant change in abundance between the VPA-resistant epilepsy group and VPA-sensitive epilepsy group, of which 13 were upregulated and 16 were downregulated in the VPA-resistant epilepsy group (Figures 3A,B, Supplementary Table S3).

To obtain a more comprehensive and in-depth understanding of differentially expressed proteins (DEPs), functional annotation using the GO database and enrichment of signaling pathways using the KEGG database were carried out. These DEPs were particularly enriched in BP involving “localization”, “response to stimulus”, “metabolic process” and “inflammatory response”. With regard to MF, the DEPs were involved in “binding”, “protein binding”, “ion binding” and “cytokine activity”. With respect to CC, DEPs were principally enriched in “cell part”, “intracellular”, “intracellular part” and “organelle” (Figure 3C). Furthermore, the DEPs were then subjected to enrichment analysis of signaling pathways using the KEGG database: DEPs were involved in “necroptosis”, “inflammation” and “cytokine–cytokine receptor interaction” (Figure 3D). Taken together, these results suggested that expression of inflammation-related mediators may change significantly in VPA-resistant epilepsy.

To further determine the hub DEPs between VPA-sensitive epilepsy and VPA-resistant epilepsy, a PPI network were constructed using the STRING database. To determine the module with the best connectivity, the MCODE plug-in of Cytoscape was used. Surprisingly, the top module contained HIF-1α, and IL-1β and TNF-α interacted directly with HIF-1α (Figure 3E). Therefore, HIF-1α, IL-1β, and TNF-α were selected as key proteins for subsequent research.

### Validation of Selected Proteins

**FIGURE 4:**
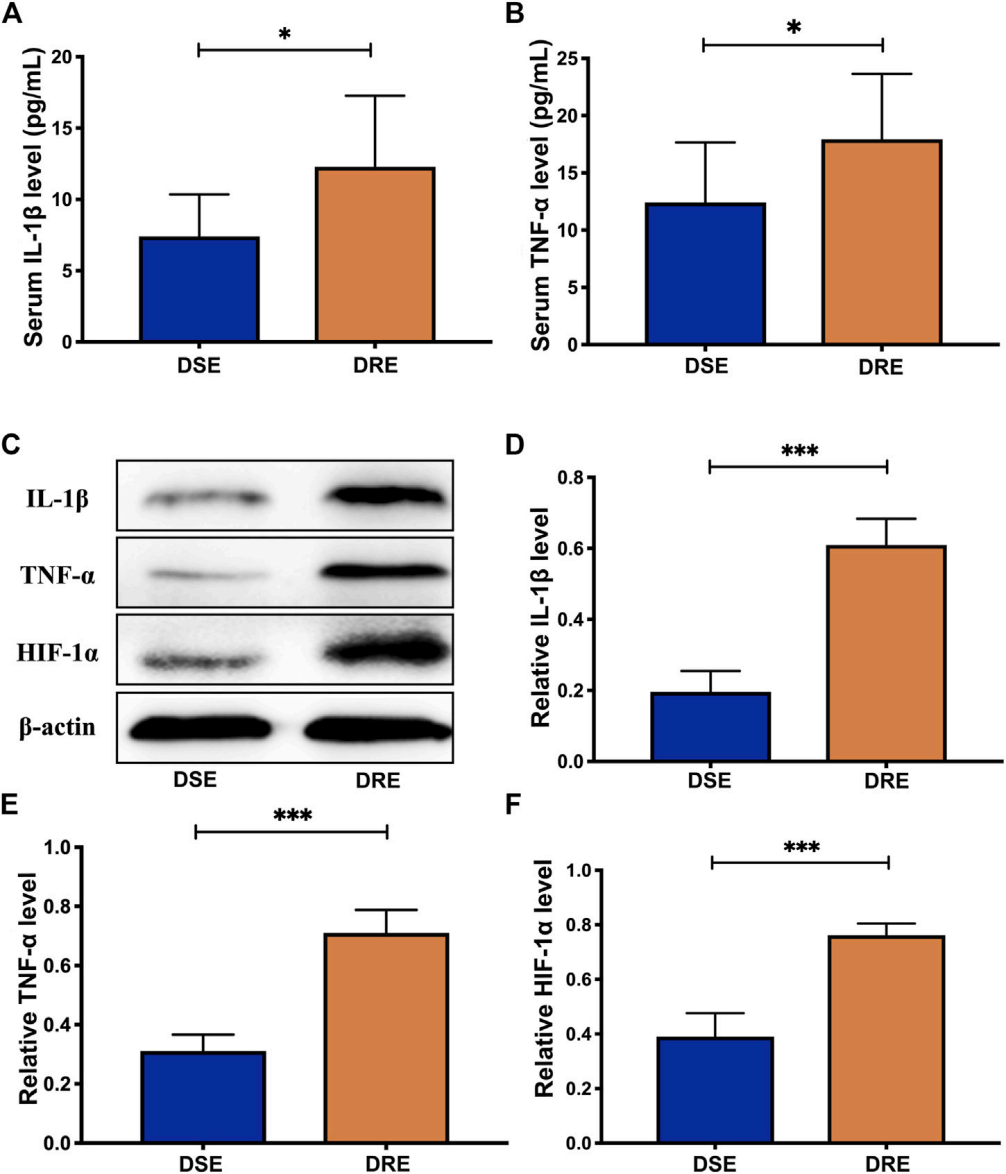
Validation analysis of selected proteins. The level of IL-1β **(Α)** and TNF-α **(Β)** in the blood of children with VPA-resistant epilepsy significantly higher than that in VPA-sensitive epilepsy using ELISA analysis (DSE: *n* = 8; DRE: *n* = 16). **(C)** Representative protein bands of IL-1β, TNF-α and HIF-1α of hippocampus determined by western blotting (*n* = 6). Protein expression of IL-1β **(D)**, TNF-α **(E)** and HIF-1α **(F)** in the hippocampus of mice with VPA-resistant epilepsy greatly higher than that of mice with VPA-sensitive epilepsy (*n* = 6). Values are the mean ± S.D. Unpaired *t*-test. *p < 0.05, **p < 0.01, and ***p < 0.001. DSE: VPA-sensitive epilepsy and DRE: VPA-resistant epilepsy.

To further verify the dysregulation of these proteins, expression of IL-1β and TNF-α in blood of children with VPA-resistant epilepsy and VPA-sensitive epilepsy was measured by ELISA. HIF-1α is an unstable macromolecular substance and cannot penetrate the blood–brain barrier, so its accumulation in the brain cannot reach the blood. Therefore, we did not measure HIF-1α expression in blood samples from children. There were no obvious differences in age, sex, course, age of onset, or seizure frequency of first 6 months of diagnosis between VPA-resistant epilepsy and VPA-sensitive epilepsy (Table 1). The level of IL-1β and TNF-α in the VPA-resistant group was substantially higher than that in children with VPA-sensitive epilepsy (Figures 4A,B). Western blotting in mice hippocampal tissue was carried out, and results indicated that expression of HIF-1α, IL-1β, and TNF-α in the hippocampus of mice with VPA-resistant epilepsy was upregulated compared with that in mice with VPA-sensitive epilepsy (Figures 4C–F). These results were consistent with the data of proteomics analysis, and support the notion that HIF-1α, IL-1β, and TNF-α showed aberrant expression in VPA-resistant epilepsy.

**TABLE 1:** Comparison of clinical data in patients with VPA-resistant epilepsy and VPA-sensitive epilepsy.

Variable	VPA-resistant epilepsy	VPA-sensitive epilepsy	*p* value
Sample size	16	8	NA
Age (years)	7.13 ± 3.76	8.25 ± 3.20	*p* > 0.05
F/M ration	9/7	4/4	*p* > 0.05
Course (years)	3.25 ± 1.53	3.75 ± 2.66	*p* > 0.05
Age of seizure onset (years)	3.88 ± 2.97	4.50 ± 2.07	*p* > 0.05
Seizure frequency (times/first 6 months of diagnosis)	26.69 ± 13.19	21.87 ± 16.11	*p* > 0.05
Seizure frequency (times/last 6 months)	13.68 ± 7.21	0	*p* < 0.05

F, female; M, male; NA, not applicable.

### Increased Activation of Microglia in VPA-Resistant Epilepsy

**FIGURE 5:**
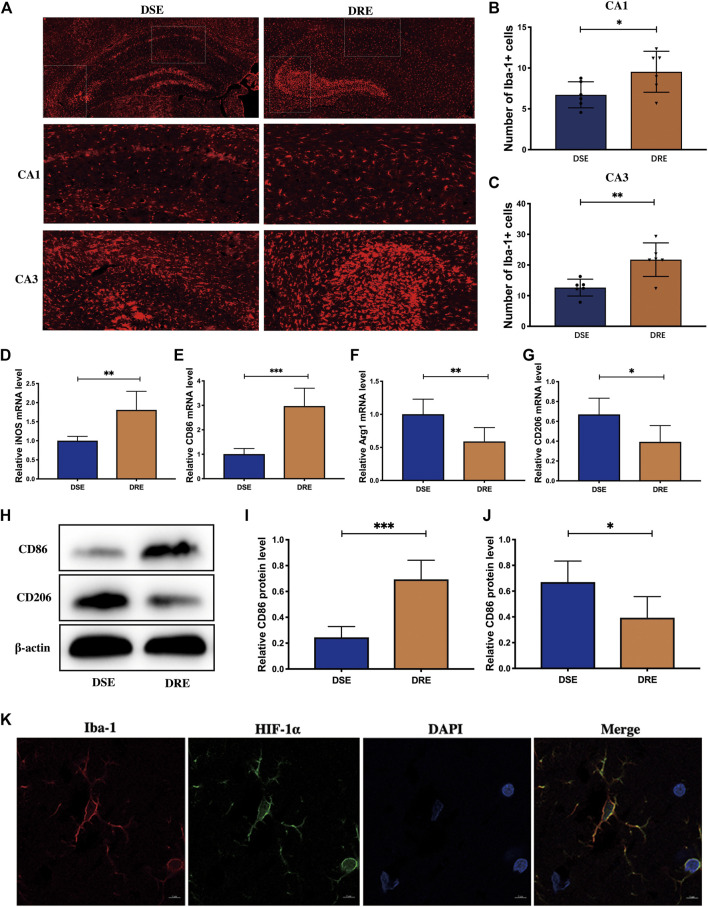
Microglia activation in the hippocampus of mice (*n* = 6). **(Α)** Representative immunohistochemistry images of Iba-1 in the hippocampus. Quantification of Iba-1-positive cells in the hippocampal CA1 **(B)** and CA3 **(C)** regions. The number of Iba-1-positive microglia in hippocampal CA1 and CA3 regions was obviously increased in VPA-resistant epilepsy. The mRNA levels of M1 subtype markers iNOS **(D)** and CD86 **(E)** are significantly increased in hippocampus of mice with VPA-resistant epilepsy. The level of M2 subtype markers Arg-1 **(F)** and CD206 **(G)** was decreased significantly in VPA-resistant epilepsy compared with VPA-sensitive epilepsy. **(H)** Representative protein bands of CD86 and CD206 of hippocampus determined by western blotting. The protein level of CD86 **(I)** was significantly decreased, and the protein level of CD208 **(J)** was significantly increased in hippocampus of VPA-resistant epilepsy mice. **(K)** Double-labeling immunofluorescence demonstrated that HIF-1α and Iba-1 co-localized in the hippocampus of mice with VPA-resistant epilepsy. Values are the mean ± S.D. Unpaired *t*-test. *p < 0.05, **p < 0.01, and ***p < 0.001. DSE: VPA-sensitive epilepsy and DRE: VPA-resistant epilepsy.

Based on the exacerbated inflammatory response in VPA-resistant epilepsy, we examined the activated status of microglia in the hippocampus of mice brain sections by immunofluorescent staining using Iba-1. The fluorescence intensity of Iba-1-labeled microglia was significantly greater in the hippocampal CA1 and CA3 regions of mice in the VPA-resistant epilepsy group compared with that in the VPA-sensitive epilepsy group (Figure 5A). The number of Iba-1-positive microglia in hippocampal CA1 and CA3 regions was obviously increased in VPA-resistant epilepsy (Figures 5B,C).

To ascertain which microglial phenotype was activated, we measured expression of M1/M2-specific markers in the hippocampus of mice using RT-qPCR. mRNA expression of iNOS and CD86 (both markers of the M1 subtype) was increased markedly in VPA-resistant epilepsy (Figures 5D,E), whereas expression of Arg-1 and CD206 (both markers of the M2 subtype) was decreased significantly in VPA-resistant epilepsy compared with VPA-sensitive epilepsy (Figures 5F,G). The trends of protein expression of CD86 and CD206 were consistent with their mRNA expression (Figures 5H–J). Collectively, our data showed polarization of microglia from the M2 phenotype to the M1 phenotype in VPA-resistant epilepsy.

Multiple studies have demonstrated that HIF-1α-related signaling pathways regulate microglia activation. Given our observation, we investigated if increased expression of HIF-1α occurs in microglia by double-labeling immunofluorescence of HIF-1α and Iba-1. HIF-1α and Iba-1 co-localized in the hippocampus of mice with VPA-resistant epilepsy (Figure 5K). This result implied that HIF-1α may regulate inflammation by activating microglia in VPA-resistant epilepsy.

### Downregulation of miR-221-3p Expression in VPA-Resistant Epilepsy

**FIGURE 6:**
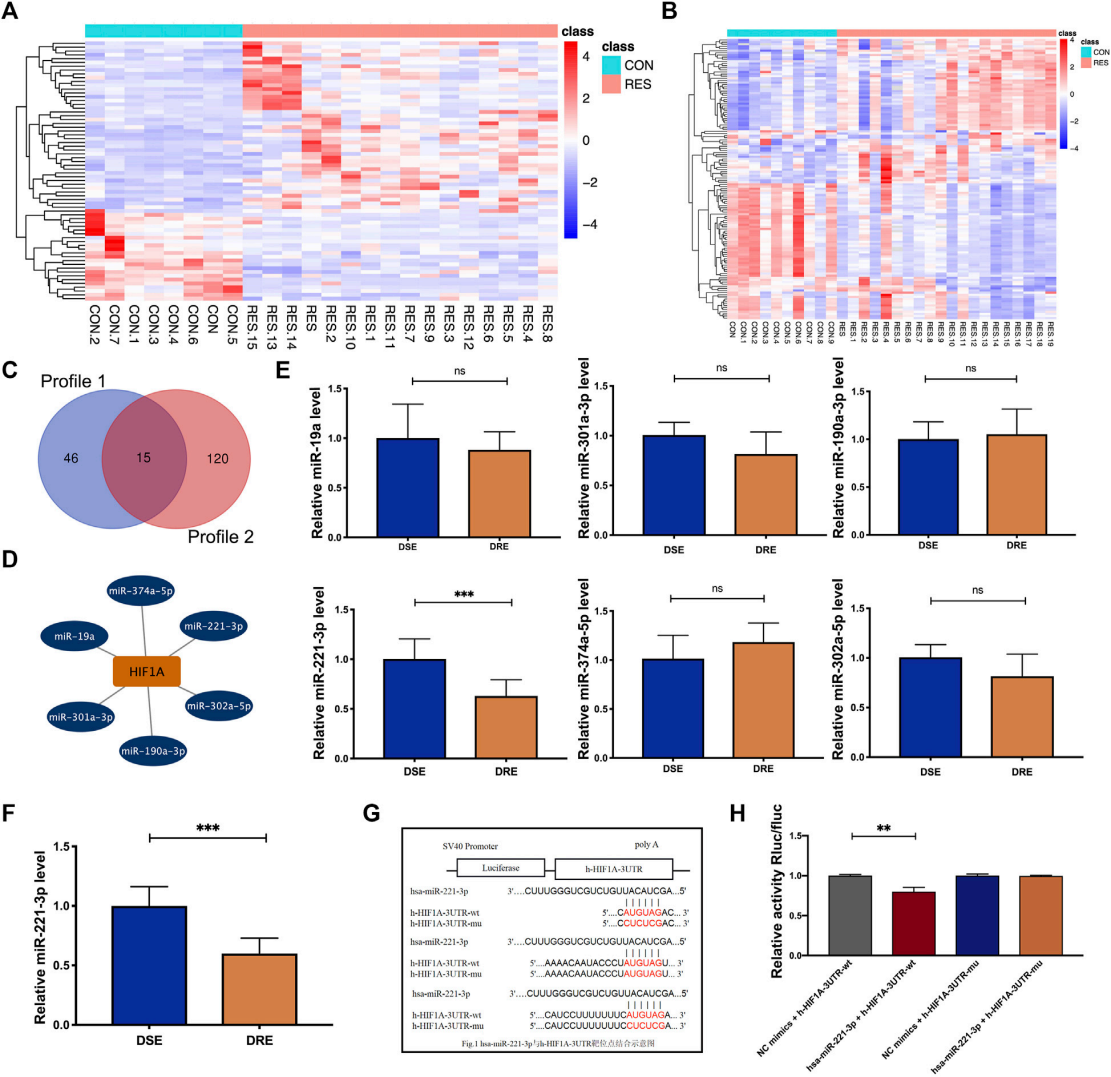
Identification of the miRNA regulating HIF-1α expression in VAP-resistant epilepsy. **(A)** Heatmap of the 71 differentially expressed miRNAs of Profile 1. **(B)** Heatmap of the 135 differentially expressed miRNAs of Profile 2. **(C)** Profile 1 and Profile 2 had 15 intersected differentially expressed miRNAs screened by a Venn diagram. **(D)** The starbase database predicted that six differentially expressed miRNAs (miR-374a-5p, miR-221-3p, miR-302a-5p, miR-190a-3p, miR-301a-3p, and miR-19a) potentially regulate the expression of HIF-1α. **(E)** MiR-221-3p was reduced significantly in the blood of children with VPA-resistant epilepsy compared with that of children with VPA-sensitive epilepsy. There is no significant difference in miR-374a-5p, miR-302a-5p, miR-190a-3p, miR-301a-3p and miR-19a between VPA-sensitive group and VPA-resistant group (DSE: *n* = 8; DRE: n = 16). **(F)** MiR-221-3p in hippocampus of VPA-resistant group was significantly lower than that in VPA-sensitive group (*n* = 6). **(G)** The predicted binding sites of miR-221-3p in the 3′-UTR of HIF-1α was verified by luciferase reporter gene assay. **(H)** Dual luciferase reporter gene assay demonstrated that miR-221-3p could negatively regulated the expression of HIF-1α. Values are the mean ± S.D. Unpaired *t*-test. *p < 0.05, **p < 0.01, and ***p < 0.001. DSE: VPA-sensitive epilepsy and DRE: VPA-resistant epilepsy. NC: negative control, Wt: wild type, and Mut: mutant.

Multiple studies have shown that miRNAs are valuable biomarkers for the diagnosis and treatment of epilepsy-related diseases. Therefore, we investigated the miRNAs that regulate HIF-1α expression in patients with VPA-resistant epilepsy. Firstly, we re-analyzed two miRNA profiles of hippocampus tissue from patients with DRE published in public databases. The difference analysis of the above two miRNA profiles between DRE and control was preformed using the “DESeq2” R package. There were 71 differentially expressed miRNAs in Profile 1 (Figure 6A) and 135 differentially expressed miRNAs in Profile 2 (Figure 6B). Profile 1 and Profile 2 had 15 intersected differentially expressed miRNAs screened by a Venn diagram (Figure 6C). The starbase database predict that miR-374a-5p, miR-221-3p, miR-302a-5p, miR-190a-3p, miR-301a-3p, and miR-19a) had potential binding sites on and the 3′-UTR sequences of HIF-1α (Figure 6D).

Subsequently, expression of these six miRNAs was measured in the plasma of children with VPA-resistant/sensitive epilepsy by RT-qPCR. However, οnly miR-221-3p was reduced significantly in the plasma of children with VPA-resistant epilepsy compared with that in children with VPA-sensitive epilepsy (Figure 6E). This result was confirmed in the hippocampal tissue of VPA-resistant mice (Figure 6F).

The predicted binding site of miR-221-3p and HIF-1α was determined using a luciferase reporter gene assay. The luciferase activity of HIF-1α-Wt in the miR-221-3p mimic group was reduced greatly compared with that in the NC group. In HIF-1α-Mut cells, there was no obvious difference between the miR-221-3p mimic group and NC group (Figures 6G,H). This result illustrated that downregulated expression of miR-221-3p in VPA-resistant epilepsy may cause its negative regulatory target gene, HIF-1α, to accumulate.

### MiR-221-3p Mimics/HIF-1α Inhibitor Could Attenuates Seizures and Inflammatory Response in VPA-Resistant Epilepsy

**FIGURE 7:**
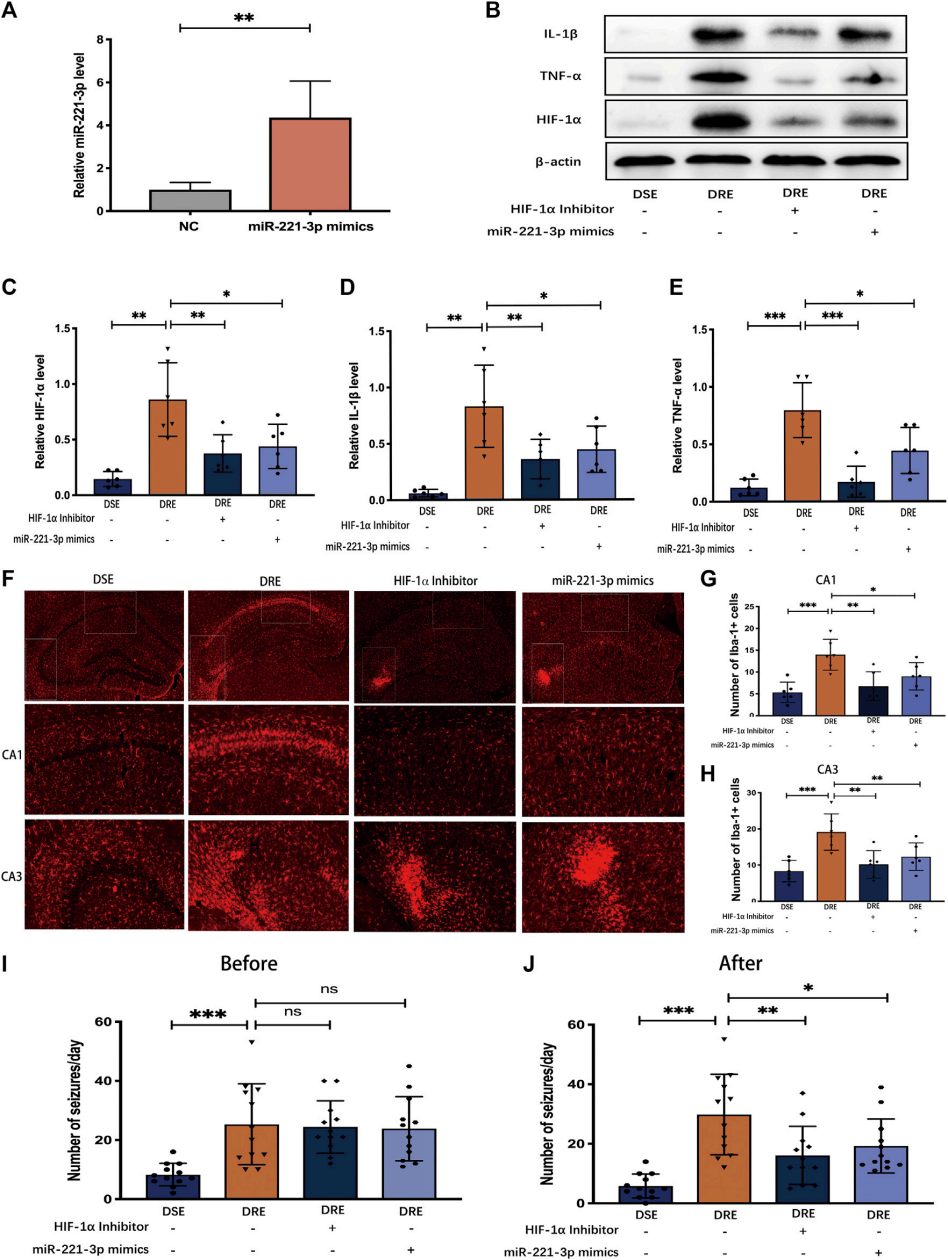
Effect of miR-221-3p mimics and HIF-1α inhibitor in mice with VPA-resistant epilepsy (*n* = 6). **(A)** MiR-221-3p mimics effectively up-regulated the level of miR-221-3p in hippocampus by 4 times. (**B)** Representative protein bands of IL-1β, TNF-α, and HIF-1α as determined by western blotting. MiR-221-3p mimics and HIF-1α inhibitor can significantly reduce the proteins expression of IL-1β **(C)** TNF-α **(D)** and HIF-1α **(E)** in the hippocampus of mice with VPA-resistant epilepsy. **(F)** Representative immunohistochemistry images of Iba-1 in the CA1 and CA3 region of hippocampus. MiR-221-3p mimics and HIF-1α inhibitor evidently decreased the number of Iba-1-positive cells in the hippocampal CA1 **(G)** and CA3 **(H)** regions. **(I)** Before intervention with miR-221-3p mimics and HIF-1α inhibitor, there were no obvious difference in the number of seizures between the VPA-resistant group with the miR-221-3p mimics or HIF-1α inhibitor group. **(J)** MiR-221-3p mimics and HIF-1α inhibitor inhibited the seizure of VPA-resistant epilepsy. Values are the mean ± S.D. Unpaired *t*-test. *p < 0.05, **p < 0.01, and ***p < 0.001. DSE: VPA-sensitive epilepsy and DRE: VPA-resistant epilepsy.

**FIGURE 8:**
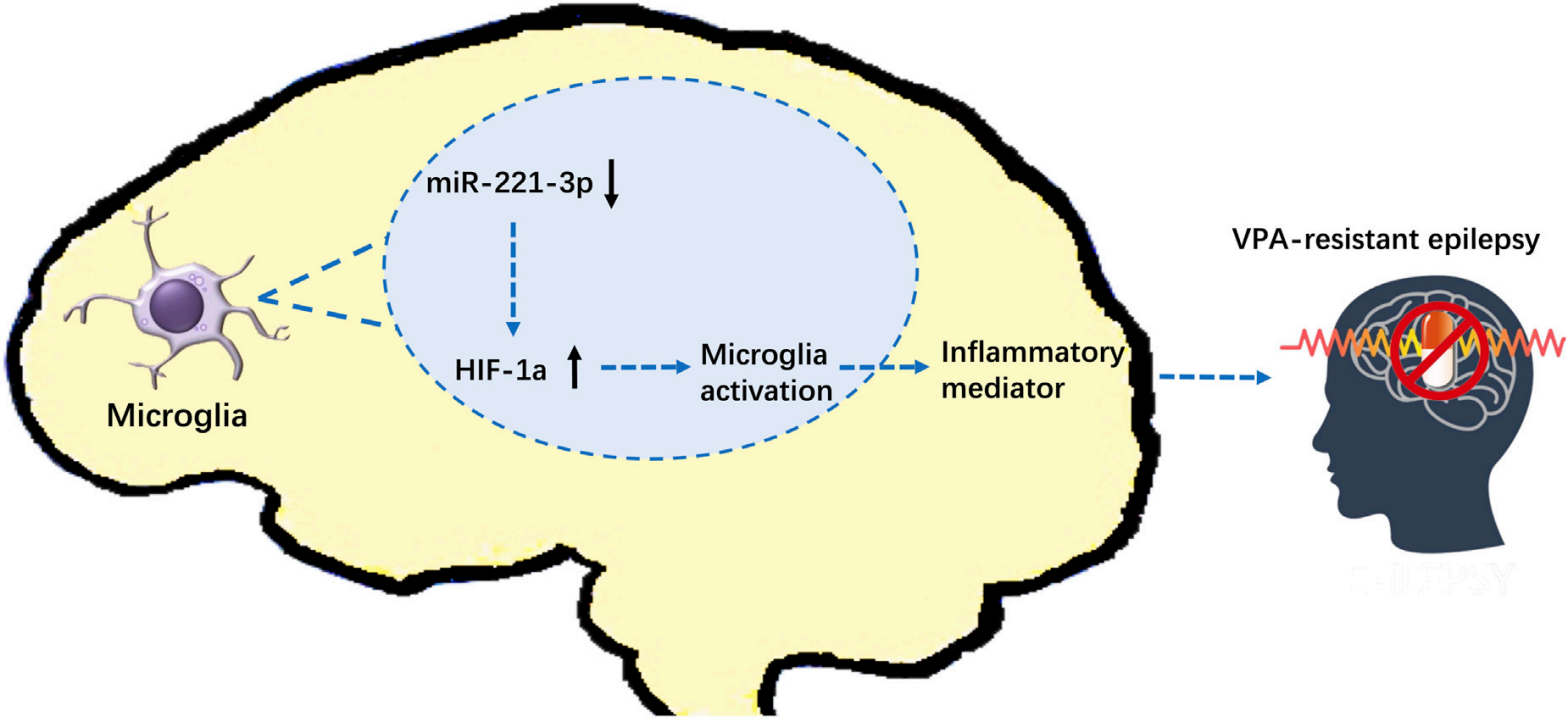
Effect of miR-221-3p/HIF-1α on VPA-resistant epilepsy (schematic). MiR-221-3p is downregulated in VPA-resistant epilepsy, which leads to the increase of its target gene: HIF-1α. Increased expression of HIF-1α activates microglia to release proinflammatory mediators, which exacerbates seizures of VPA-resistant epilepsy.

The above results indicate that down-regulated miR-221-3p and up-regulated HIF-1α may be involved in the pathogenesis of VPA-resistant epilepsy. To test this hypothesis, we surveyed the effect of miR-221-3p mimics and 2ME2 (HIF-1α inhibitor) in VPA-resistant epilepsy. First, we demonstrated that miR-221-3p mimics markedly increased miR-221-3p expression and 2ME2 evidently reduced the expression of HIF-1α in the hippocampus of mice with VPA-resistant epilepsy (Figures 7A,B). Subsequently, we confirmed that up-regulated miR-221-3p significantly reduce HIF-1α expression in the hippocampus of mice with VPA-resistant epilepsy, which reduced expression of IL-1β and TNF-α further (Figure 7 B ∼ E). MiR-221-3p mimics or 2ME2 could reduce the number of microglia activated in the CA1 and CA3 regions of the hippocampus in mice with VPA-resistant epilepsy (Figures 7F–H). Meanwhile, miR-221-3p mimics or 2ME2 could decreased the number of increased seizures in VPA-resistant epilepsy (Figures 7I,J). Taken together, these data showed that increasing miR-221-3p expression reduced HIF-1α expression. This action decreased expression of proinflammatory factors and the number of activated microglia and, ultimately, reduced the number of seizures in mice with VPA-resistant epilepsy (Figure 8).

## Discussion

We investigated the effect of HIF-1α in VPA-resistant epilepsy. It is a key regulator of local hypoxia and neuroinflammation, which are important clinical phenomena in VPA-resistant epilepsy. The role of HIF-1α in the mechanism of VPA-resistant epilepsy is still unclear or even controversial. We showed that downregulation of miR-221-3p expression in VPA-resistant epilepsy led to accumulation of its negative regulatory target gene: HIF-1α. These actions led to the activation of mocroglia, accompanied by upregulation of proinflammatory mediator and aggravation epileptic behavior. This is the first study to demonstrate the significance of miR-221-3p/HIF-1α in VPA-resistant epilepsy.

An increasing body of evidences suggests that inflammatory mediators are widely involved in, and sufficient for, generating epileptic seizures. Anti-inflammatory compounds, such as IRL-1 blockers and COX-2 inhibitors (Citraro et al., 2015), can significantly suppress the development of spontaneous recurrent seizures and reduce the extent of CA1 injury and sprouting of mossy fibers (Aronica et al., 2017). Previously, we showed that IL-1β, TNF-α, and other proinflammatory mediators are overexpressed in VPA-resistant epilepsy, but we did not explore the mechanisms that trigger this phenomenon.

To address this question, we established VPA-resistant mice and VPA-sensitive mice. Proteomics analysis revealed that overexpressed HIF-1α interacted with IL-1β and TNF-α in mice with VPA-resistant epilepsy, which was also confirmed by western blotting. The regulatory effect of HIF-1α on IL-1β expression has been studied in the context of sarcoidosis, infections, and cancer. Studies have shown that HIF-1 as a transcription factor be composed of HIF-1α and HIF-1β subunits, which translocate into the nucleus and bind to hypoxia-response elements to regulate the transcriptional expression of IL-1β. Studies have also shown that HIF-1α is responsible for recruiting M1 macrophages that release TNF-α in patients with heart failure (Warbrick and Rabkin, 2019). Above results evidence that upregulation of HIF-1α expression in VPA-resistant epilepsy leads to increased expression of IL-1β and TNF-α.

Prior has indicated that excessive activation of microglia in patients with epilepsy exacerbates the release of proinflammatory mediators (Patel et al., 2019). Activated microglia release proinflammatory cytokines, which can lead to neuronal hyperexcitability. In addition, microglia induced disruptions in neuronal circuits by impairing synaptic pruning, which leads to seizures (Schafer et al., 2016). Boer et al. found that patients with focal cortical dysplasia (a major cause of DRE) have a specific and persistent increase in the number of microglia within the dysplastic region (Boer et al., 2006). Our results indicated that the number of microglia in the CA1 and CA3 regions of the hippocampus of mice with VPA-resistant epilepsy was increased greatly, and was accompanied by microglial polarization from the M2 phenotype to the M1 phenotype. Microglial activation in the CNS is heterogeneous, and is categorized by the M1 phenotype (pro-inflammation) and M2 phenotype (anti-inflammation) (Lan et al., 2017). The dynamic changes of microglia from the M2 subtype to M1 subtype is a hallmark of the inflammatory response of the CNS (Qiu et al., 2020). Studies have shown that HIF-1α related signaling pathways regulate microglia activation in Alzheimer’s disease (Baik et al., 2019). Han et al. indicated that HIF-1α deficient glial reduced neutrophil migration and infarct, which suppressed the release of IL-1β and CXCL1. Therefore, we explored the localization of HIF-1α and microglia. The results are consistent with this point. HIF-1α was co-localized with microglia markers. That is, The accumulation of HIF-1α in VPA-resistant epilepsy caused activation of microglia and aggravated the release of proinflammatory mediators.

Biomarkers for identification and treatment of VPA-resistant epilepsy are crucial for tailored treatment and optimal allocation of healthcare resources for this patient population (Kalilani et al., 2018). As a small molecule, most of miRNAs can penetrate the blood-brain barrier and exist stably in blood and urine. Therefore, we investigated specific miRNAs in VPA-resistant epilepsy, hoping that it can become a valuable biomarker. As we know, it is difficult to collect hippocampal tissue from children with VPA-resistant epilepsy in clinical practice. In order to efficiently find the miRNA that regulates HIF-1α in VPA-resistant epilepsy, this study firstly performed integrated bioinformatics analysis on the hippocampal tissue of patients with drug-resistant epilepsy in public databases referring to the previous method (Xu et al., 2020). Subsequently, the miRNAs that potentially regulate the expression of HIF-1α were selected. Finally, these miRNAs were screened in the blood samples of children with VPA-resistant epilepsy, and were verified in the hippocampus of VPA-resistant epilepsy mice. We identified that miR-221-3p was down-regulated markedly in VPA-resistant epilepsy, and negatively regulated the expression of the target gene: *HIF-1α*. The regulatory effect of miR-221-3p on the expression of HIF-1α is consistent with a recently published study, which demonstrated that HIF-1α was the target gene of miR-221-3p in patients with heart failure and antagomiR-221-3p increased HIF-1α expression (Li et al., 2021). Subsequently, we demonstrated that miR-221-3p mimics effectively reduce the expression of HIF-1α in the hippocampus of mice with VPA-resistant epilepsy, thereby reducing the number of microglia, inhibiting the release of proinflammatory mediators, and alleviating the number of seizures markedly.

However, our present work still has some limitations. Firstly, in clinical practice, multi-drug combination therapy is the main approach for children with epilepsy. Therefore, the children with VPA-resistant epilepsy enrolled into this study were resistant to multiple AEDs. Since multiple AEDs are involved in the neuroinflammatory process (Itoh et al., 2019; Park et al., 2020), the resistance to other AEDs may be a confounding factor in our study. In future studies, we will further investigate the significance of neuroinflammatory processes in VPA-resistant epilepsy, while considering the effect of other common antiepileptic drugs. Secondly, various inflammatory mediators such as CXCL9, CXCL3, and IL-6 have been shown to be dysregulated in patients with drug-resistant epilepsy. Since this study focused on the role of HIF-1α in VPA-resistant epilepsy, we prioritized IL-1β and TNF-α that directly interact with HIF-1α. In future study, we will pay attention to the effects of chemokines and cytokines released by activated microglia in VPA-resistant epilepsy. Thirdly, this study found the dysregulation of miR-221-3p, IL-1β, and TNF-α in VPA-resistant epilepsy, and confirmed the influence of these mediators on seizure severity. However, due to the small number of patients enrolled, we have not performed further studies on the correlation between these mediators and clinical parameters.

## Conclusion

We demonstrated that expression of miR-221-3p, as a potential biomarker, is downregulated in VPA-resistant epilepsy, which leads to increase in expression of its target gene: HIF-1α. Increased expression of HIF-1α activates microglia to release proinflammatory mediators, which exacerbates seizures. Our data suggest the effect of miR-221-3p/HIF-1α on VPA-resistant epilepsy. These alterations should be considered when studying the mechanisms of VPA-resistant epilepsy and designing drug treatments.

## Data Availability

The datasets presented in this study can be found in online repositories. The names of the repository/repositories and accession number(s) can be found in the article/Supplementary Material
