# Supplementary material for: MicroRNA-221-3p Suppresses the Microglia Activation and Seizures by Inhibiting of HIF-1α in Valproic Acid-Resistant Epilepsy
Source: Front Pharmacol. 2021 Aug 23;12:714556. doi: 10.3389/fphar.2021.714556 (PMC8419275; doi:10.3389/fphar.2021.714556)
Supplement: Supplementary file 1 [file Table1.docx]

**Supplemental Table 1**

Clinical materials of the VPA-resistant epilepsy patients in the study.

| Patients No. | Age (years) | Gender | AEDs | Course (Years) | Age of seizure onset  (years) | Seizure frequency  (times/first 6 months of diagnosis) | Seizure frequency  (times/last 6 months) |
| --- | --- | --- | --- | --- | --- | --- | --- |
| 1 | 4 | Female | VPA, OXC, LTG, PB | 2 | 2 | 23 | 12 |
| 2 | 5 | Female | VPA, LEV | 2 | 3 | 27 | 9 |
| 3 | 10 | Female | VPA, OCX, LEV | 6 | 4 | 36 | 12 |
| 4 | 14 | Female | VPA, TPM, LTG, CLZ | 4 | 10 | 21 | 6 |
| 5 | 5 | Female | VPA, TPM, LEV | 2 | 3 | 51 | 17 |
| 6 | 5 | Female | VPA, OXC, LEV | 2 | 3 | 45 | 28 |
| 7 | 3 | Female | VPA, TPM, LEV | 2 | 1 | 48 | 27 |
| 8 | 14 | Female | VPA, TPM, LTG, LEV | 5 | 9 | 21 | 16 |
| 9 | 5 | Female | VPA, TPM, LEV | 2 | 3 | 23 | 7 |
| 10 | 4 | Male | VPA, TPM, GVB | 4 | <1 | 19 | 5 |
| 11 | 10 | Male | VPA, TPM, LEV | 2 | 8 | 11 | 6 |
| 12 | 6 | Male | VPA, LEV, CBZ | 5 | 1 | 12 | 8 |
| 13 | 5 | Male | VPA, LEV | 2 | 3 | 9 | 13 |
| 14 | 5 | Male | VPA, LEV, LTG | 3 | 2 | 15 | 19 |
| 15 | 6 | Male | VPA, OCX, TPM | 3 | 3 | 32 | 21 |
| 16 | 13 | Male | VPA, OCX, LEV | 6 | 7 | 34 | 13 |

AEDs, anti-epileptic drugs; VPA, valproic; OXC, oxcarbazepine; LTG, lamotrigine; PB, Phenobarbital; LEV, levetiracetam; TPM, topiramate.
