# Supplementary material for: MicroRNA-221-3p Suppresses the Microglia Activation and Seizures by Inhibiting of HIF-1α in Valproic Acid-Resistant Epilepsy
Source: Front Pharmacol. 2021 Aug 23;12:714556. doi: 10.3389/fphar.2021.714556 (PMC8419275; doi:10.3389/fphar.2021.714556)
Supplement: Supplementary file 2 [file Table2.docx]

**Supplemental Table 2**

Clinical materials of the VPA-sensitive epilepsy patients in the study.

| Patients No. | Age (years) | Gender | AEDs | Course (Years) | Age of seizure onset (years) | | Seizure frequency  (times/first 6 months of diagnosis) | | Seizure frequency (times/last 6 months) |
| --- | --- | --- | --- | --- | --- | --- | --- | --- | --- |
| 1 | 4 | Female | VPA | 3 | 1 | 12 | | 0 | |
| 2 | 9 | Female | VPA, OCX | 6 | 3 | 23 | | 0 | |
| 3 | 14 | Female | VPA, TPM | 9 | 5 | 11 | | 0 | |
| 4 | 6 | Female | VPA, LTG | 1 | 5 | 32 | | 0 | |
| 5 | 7 | Male | VPA | 2 | 5 | 9 | | 0 | |
| 6 | 9 | Male | VPA, LEV | 2 | 7 | 56 | | 0 | |
| 7 | 6 | Male | VPA, TPM | 3 | 3 | 23 | | 0 | |
| 8 | 11 | Male | VPA | 4 | 7 | 9 | | 0 | |

AEDs, anti-epileptic drugs; VPA, valproic; OXC, oxcarbazepine; LTG, lamotrigine; LEV, levetiracetam; TPM, topiramate.
